# Validity of the CR-POSSUM model in surgery for colorectal cancer in Spain (CCR-CARESS study) and comparison with other models to predict operative mortality

**DOI:** 10.1186/s12913-018-2839-x

**Published:** 2018-01-29

**Authors:** Marisa Baré, Manuel Jesús Alcantara, Maria José Gil, Pablo Collera, Marina Pont, Antonio Escobar, Cristina Sarasqueta, Maximino Redondo, Eduardo Briones, Paula Dujovne, Jose Maria Quintana, on behalf of the CARESS-CCR Study Group, Maximino Redondo, Francisco Rivas, Eduardo Briones, Elena Campano, Ana Isabel Sotelo, Francisco Medina, Arturo Del Rey, Maria M. Morales, Segundo Gómez, Marisa Baré, Marina Pont, Núria Torà, Manuel Jesús Alcántara, Maria José Gil, Miquel Pera, Pablo Collera, Josep Alfons Espinàs, Mireia Espallargues, Caridad Almazán, Mercè Comas, Nerea Fernández, Juan Antonio Blasco, Isabel del Cura, Paula Dujovne, José María Fernández, Rocío Anula, Julio Ángel Mayol, Ramón Cantero, Héctor Guadalajara, María Heras, Damián García, Mariel Morey, José María Quintana, Nerea González, Susana García, Iratxe Lafuente, Urko Aguirre, Miren Orive, Josune Martin, Ane Antón, Santiago Lázaro, Cristina Sarasqueta, Jose María Enriquez, Carlos Placer, Amaia Perales, Antonio Escobar, Amaia Bilbao, Alberto Loizate, Inmaculada Arostegui, José Errasti, Iñaki Urkidi, Jose María Erro, Enrique Cormenzana, Antonio Z. Gimeno

**Affiliations:** 1 Clinical Epidemiology and Cancer Screening, Parc Taulí Sabadell-University Hospital, Parc Taulí 1, 08208 Sabadell, Spain; 2 Obstetrics, Gynecology and Preventive Medicine Department, Autonomous University of Barcelona-UAB, Cerdanyola del Vallès, Spain; 3 Coloproctology Unit, General and Digestive Surgery Service, Parc Taulí Sabadell- University Hospital, Sabadell, Spain; 4 General and Digestive Surgery Service, Parc de Salut Mar, Barcelona, Spain; 5 General and Digestive Surgery Service, Althaia - Xarxa Assistencial Universitaria, Manresa, Spain; 6 Research Unit, Hospital Universitario Basurto, Bilbao, Spain; 7 Unidad de Investigación, Hospital Universitario Donostia/Instituto de Investigación Sanitaria Biodonostia, Donostia, Spain; 8 Research Unit, Agencia Sanitaria Costa del Sol, Marbella, Spain; 9 Unidad de Epidemiología. Distrito Sevilla, Servicio Andaluz de Salud, Sevilla, Spain; 10 Servicio de Cirugía General y del Aparato Digestivo, Hospital Universitario Fundación Alcorcón, Madrid, Spain; 11 Research Unit, Hospital Galdakao-Usansolo, Galdakao, Spain; 12 Health Services Research on Chronic Patients Network, Sabadell, Spain

**Keywords:** Operative mortality, Colorectal cancer, Predictive model, Cr-possum

## Abstract

**Background:**

To validate and recalibrate the CR- POSSUM model and compared its discriminatory capacity with other European models such as POSSUM, P-POSSUM, AFC or IRCS to predict operative mortality in surgery for colorectal cancer.

**Methods:**

Prospective multicenter cohort study from 22 hospitals in Spain. We included patients undergoing planned or urgent surgery for primary invasive colorectal cancers between June 2010 and December 2012 (*N* = 2749). Clinical data were gathered through medical chart review. We validated and recalibrated the predictive models using logistic regression techniques. To calculate the discriminatory power of each model, we estimated the areas under the curve - AUC (95% CI). We also assessed the calibration of the models by applying the Hosmer-Lemeshow test.

**Results:**

In-hospital mortality was 1.5% and 30-day mortality, 1.7%. In the validation process, the discriminatory power of the CR-POSSUM for predicting in-hospital mortality was 73.6%. However, in the recalibration process, the AUCs improved slightly: the CR-POSSUM reached 75.5% (95% CI: 67.3–83.7). The discriminatory power of the CR-POSSUM for predicting 30-day mortality was 74.2% (95% CI: 67.1–81.2) after recalibration; among the other models the POSSUM had the greatest discriminatory power, with an AUC of 77.0% (95% CI: 68.9–85.2). The Hosmer-Lemeshow test showed good fit for all the recalibrated models.

**Conclusion:**

The CR-POSSUM and the other models showed moderate capacity to discriminate the risk of operative mortality in our context, where the actual operative mortality is low. Nevertheless the IRCS might better predict in-hospital mortality, with fewer variables, while the CR-POSSUM could be slightly better for predicting 30-day mortality.

**Trail registration:**

Registered at: ClinicalTrials.gov Identifier: NCT02488161

**Electronic supplementary material:**

The online version of this article (10.1186/s12913-018-2839-x) contains supplementary material, which is available to authorized users.

## Background

Colorectal cancer is one of the most common cancers in developed countries; in Europe alone, more than 340,000 people were diagnosed in 2012, and the incidence is increasing in many countries [1]. The mainstay of treatment is surgery, whether to resect the tumor and/or its metastases or to alleviate symptoms of the disease [2]. Surgery for colorectal cancer is highly complex and involves significant risks that can lead to unfavourable short-term outcomes. Operative mortality (death after surgery before discharge from hospital or within 30 days of surgery) is a quality indicator for surgery, because of its relationship with preoperative preparation and the quality of postoperative care, so it is of the utmost importance to have explicit criteria to know which patients require stricter surveillance.

Various authors have developed predictive models to estimate the adjusted risk of death after a surgical intervention; these models are based on a set of variables (4–18, depending on the model) related to the patients themselves, to their disease, and/or to the surgical process. Some of these models can be applied to any surgical patient, whereas others are specific to a particular type of surgery. The Physiological and Operative Severity Score for the enUmeration of Mortality and Morbidity [3] (POSSUM) and a modified version of this score, the Portsmouth-POSSUM [4] (P-POSSUM), are examples of models applicable to any surgical patient, whereas the Colorectal POSSUM (CR-POSSUM) is a version with fewer variables that is specific for patients undergoing colorectal surgery [5].

The CR-POSSUM was first published in 2004. It comprises 10 variables, and the weights assigned to these variables make it possible to calculate a physiologic component and an intervention component, which in turn make it possible to use logistic regression to estimate the expected probability of death [5]. These models have been validated in some developed countries; although their overall discriminatory capacity is acceptable, they tend to overestimate the risk of mortality in low risk patients [6]. In the recent years, other simpler models have been developed in Europe: The model elaborated by the Association Française de Chirurgie (AFC) to predict in-hospital mortality in colorectal surgery consists of only four variables [7], and the recently published and externally validated Identification of Risk in Colorectal Surgery (IRCS) score consists of five variables [8].

A good predictive model should be feasible (the variables should be measurable before surgery), simple, and able to discriminate or identify outcomes accurately. To date, although some of these models have been validated in the countries where they were devised or in other developed countries, there is no consensus about the most appropriate instrument for predicting the risk of operative mortality. In Spain, surgery for colorectal cancer is done both at smaller, local hospitals with relatively small volumes of surgical interventions and at larger, referral hospitals with large volumes of surgical interventions. Although estimations of some quality and outcome indicators for colorectal cancer surgery at a local level have been published in Spain [9–11], and although some departments of surgery in our setting used the POSSUM models for clinical purposes until we initiated this coordinated study in 2009, there had been no validation of those models in our context and neither no predictive model had been generally adopted by surgeons to guide clinical decision making. Because the variables in the CR-POSSUM and the other POSSUM models include those variables that are considered in the IRCS and the AFC models, we thought appropriate to validate also the IRCS and AFC models in Spain.

Thus, we aimed to estimate the operative mortality in surgery for colorectal cancer in Spain, to validate and recalibrate the CR- POSSUM model in the Spanish context, and to compare its discriminatory capacity with that of other models developed in Europe to predict operative mortality in surgery for colorectal cancer.

## Methods

### Design, setting, and patients

This prospective multicenter cohort study of patients from 22 hospitals located in different areas in Spain was done in the context of the REDISSEC (Health Services Research on Chronic Diseases Network)/CCR-CARESS (Colorectal Cancer Health Services Research) study, which addressed diverse research objectives in healthcare centres treating colorectal cancer in Spain. All the hospitals provided services for the National Health System, and their size, location and level of technology varied [12]. The Clinical Research Ethics Committees of the Parc Taulí Sabadell-University Hospital; Hospital del Mar; Fundació Unió Catalana d’Hospitals; Gipuzkoa Health Area; Basque Country (CEIC-E); Hospital Galdakao-Usansolo; Hospital Txagorritxu; Hospital Basurto; La Paz University Hospital; Fundación Alcorcón University Hospital; Hospital Universitario Clínico San Carlos (formerly Clinical Research Ethics Committee of Area 7 – Hospital Clínico San Carlos); Costa del Sol Health Agency and the Regional Committee of Clinical Trials of Andalusia approved the study, and all patients provided written informed consent.

We included patients undergoing scheduled or urgent surgery for primary invasive colorectal cancers in the period comprising June 2010 through December 2012, whether the goal of surgery was to excise the tumor or to palliate symptoms.

The CCR-CARESS study, excluded patients considered by the attending physician to be in very poor overall condition or have a very limited life expectancy; those who declined to participate or did not sign the consent form; those with only cancer in situ; those with relapsed tumors; those with cancer not located in the colon or rectum; those who died before the intervention; those with inoperable cancer; those transferred for surgery in another centre; and others (e.g., language problems).

### Variables and data collection

Clinical data was gathered from clinical records or from the surgeons of the team. The variables analyzed were a) baseline characteristics such as age, sex, tumor location (colon or rectum and the distance at the anal margin), neurological comorbidities (dementia, cerebrovascular disease, hemiplegia), weight loss > 10% in 6 months and, clinical or pathological staging according to Dukes and TNM [13]. b) preoperative variables such as laboratory parameters (urea (mmol/l), haemoglobin (g/dL), leucocytes (× 10^12/l), sodium (mmol/l), potassium (mmol/l)), heart rate (beats/min), systolic blood pressure [SBP] (mmHg), heart failure (none, mild, moderate, or severe), signs of respiratory failure (no dyspnoea, dyspnoea on exertion, limiting dyspnoea, dyspnoea at rest), electrocardiogram (normal, atrial fibrillation [AF], other abnormal rhythm), and level of consciousness according to the Glasgow Coma Score. c) surgical process variables such as urgency of the intervention (scheduled, urgent, or, when done < 2 h after presentation at the emergency department, emergency), operative severity according to the National Institute for Health and Care Excellence [NICE] clinical guidelines (moderate, major or complex major) [14], tumor resection (yes or no), number of distinct surgical procedures in the same intervention (including tumor excision, ostomy, or surgery on other organs), peritoneal contamination (none, serous fluid, local pus, free pus or faeces or blood), and total blood loss (ml).

All patients were followed up after the intervention to estimate two types of operative mortality: in-hospital mortality, defined as death during the hospital stay, regardless of the length of stay, and 30-day mortality, defined as death within 30 days of the intervention, whether occurring in the hospital or after discharge.

### Models for predicting the risk of death

Table 1 lists the five models chosen to predict operative mortality, and Additional file 1: Appendix A shows the logistic regression formula used in each of them to estimate the probability of death. All the models were elaborated from some of the variables discussed above plus an ‘intercept’. The POSSUM and P-POSSUM models estimate a physiological score and an operative severity score from 18 variables; each individual’s score is calculated by summing his or her values for each variable after weighting. Finally, each score is introduced into the model and is then multiplied by its corresponding β coefficient. The CR-POSSUM, the version specific for colorectal surgery, includes only 10 variables, but the formula for calculating the score is similar. The AFC model does not involve a mathematical equation or any weighting: it consists of 4 variables that are introduced into a regression model [7]. The IRCS comprises 5 variables, each of which has a weight for each category and is multiplied by the equation’s β coefficient [8].

**Fig. 1:**
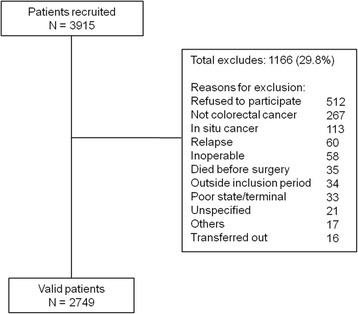
Sample size and exclusion criteria

**Table 1:** Review of scoring systems validated

Model	Year of publication	Country of development	Population	Number of model parameters	Validation	AUC [range] (Number of studies)
POSSUM	1991	United Kingdom	General surgery	18	External	[62.7–81.0] (10)
P-POSSUM	1996	United Kingdom	General surgery	18	External	[64.8–86.8] (13)
CR-POSSUM	2004	United Kingdom	Colorectal surgery	10	External	[59.0–89.8] (22)
AFC	2005	France	Colorectal surgery for malignant or diverticular disease	4	External	[71.4–89.0] (6)
IRCS	2014	Netherlands, Spain	Colorectal surgery	5	External	[83.0–85.0] (2)

*AUC* Area under the curve

**Table 2:** Univariate and Bivariate analysis of CR-POSSUM factors for operative mortality

			Total (*N* = 2749)		In-hospital mortality (*N* = 41)			30-day mortality (*N* = 47)		
	Weight		N	% Col	N	% Row	*p*-value	N	% Row	*p*-value
Age	1	<=60	640	23.3	2	0.3	< 0.001	2	0.3	< 0.001
	3	61–70	830	30.2	7	0.8		10	1.2	
	4	71–80	902	32.9	14	1.6		16	1.8	
	8	> = 81	373	13.6	18	4.8		19	5.1	
		*missing*	*4*	*0.1*						
Heart failure	1	None or mild	2487	92.9	31	1.2	< 0.001	36	1.4	< 0.001
	2	Moderate	144	5.4	6	4.2		7	4.9	
	3	Severe	45	1.7	3	6.7		3	6.7	
		*missing*	*73*	*2.7*						
Systolic blood pressure (mmHg)	1	100–170	2452	93.1	34	1.4	0.004	39	1.6	0.007
	2	> 170 or 90–99	161	6.1	4	2.5		5	3.1	
	3	< 90	20	0.8	2	10.0		2	10.0	
		*missing*	*116*	*4.2*						
Heart rate (beats/min)	1	40–100	2516	96.6	36	1.4	0.186	42	1.7	0.289
	2	101–120	76	2.9	3	3.9		3	3.9	
	3	> 120 or < 40	13	0.5	0	0.0		0	0.0	
		*missing*	*144*	*5.2*						
Urea (mmol/l)	1	<=10.0	767	31.1	14	1.8	0.440	15	2.0	0.363
	2	10.1–15.0	1005	40.8	12	1.2		15	1.5	
	3	> 15.0	694	28.1	13	1.9		17	2.4	
		*missing*	*283*	*10.3*						
Haemoglobin (g/dl)	1	13.0–16.0	1053	39.1	13	1.2	0.023	12	1.1	0.002
	2	10.0–12.9 or 16.1–18.0	1290	47.9	16	1.2		21	1.6	
	3	< 10.0 or > 18.0	350	13.0	11	3.1		14	4.0	
		*missing*	*56*	*2.0*						
Physiological score: in-hospital mortality			mean:	std. dev:	median:	min:	max:	*missing:*		
		No	10.4	2.6	10.0	6.0	19.0	*453*		
		Yes	12.9	2.9	13.0	6.0	19.0	*3*		
Operative severity	1	Minor	0	0.0	0	0.0	0.003	0	0.0	< 0.001
	3	Moderate	60	2.2	4	6.7		5	8.3	
	4	Major	1520	55.4	19	1.3		21	1.4	
	8	Complex major	1164	42.4	18	1.5		21	1.8	
		*missing*	*5*	*0.2*						
Peritoneal contamination	1	None or serous fluid	2683	98.0	37	1.4	0.001	42	1.6	< 0.001
	2	Local pus	9	0.3	1	11.1		1	11.1	
	3	Free pus or faeces or blood	46	1.7	3	6.5		4	8.7	
		*missing*	*11*	*0.4*						
Operative urgency	1	Scheduled	2649	96.4	34	1.3	< 0.001	38	1.4	< 0.001
	3	Urgent	93	3.4	6	6.5		8	8.6	
	8	Emergency	7	0.3	1	14.3		1	14.3	
		*missing*	*0*	*0.0*						
Clinical or pathological cancer staging	1	Dukes’ A-B	1582	57.9	20	1.3	0.484	21	1.3	0.003
	2	Dukes’ C	895	32.7	16	1.8		15	1.7	
	3	Dukes’ D	256	9.4	5	2.0		11	4.3	
		*missing*	*16*	*0.6*						
Operative severity score: in-hospital mortality			mean:	std. dev:	median:	min:	max:	*missing:*		
		No	9.3	2.2	8.0	6.0	20.0	*453*		
		Yes	10.0	2.5	11.0	6.0	16.0	*3*

**Fig. 2:**
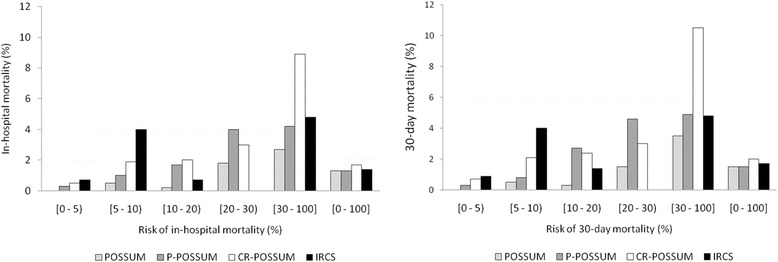
Operative death estimated by POSSUM, P-POSSUM, CR-POSSUM and IRCS

**Table 3:** External validation and recalibration of models predicting in-hospital and 30-day mortality

		In-hospital mortality					30-day mortality				
			Validation		Recalibration			Validation		Recalibration	
Model	N	Deaths included	AUC (95% CI)	H-L p- value	AUC (95% CI)	H-L p-value	Deaths included	AUC (95% CI)	H-L p-value	AUC (95% CI)	H-L p-value
POSSUM	2119	27/41	74.8 (65.6–84.0)	< 0.001	^a^75.6 (66.3–84.9)	0.548	32/47	76.3 (68.2–84.3)	< 0.001	^a^77.0 (68.9–85.2)	0.250
P-POSSUM	2119	27/41	75.7 (66.4–85.0)	< 0.001				77.1 (68.9–85.2)	< 0.001		
CR-POSSUM	2293	38/41	73.6 (65.2–82.1)	< 0.001	75.5 (67.3–83.7)	0.261	45/47	73.2 (65.9–80.6)	< 0.001	74.2 (67.1–81.2)	0.463
AFC	1465	23/41	NA	NA	68.2 (56.6–79.7)	0.994	26/47	NA	NA	68.6 (58.0–79.1)	0.984
IRCS	2653	38/47	74.4 (65.8–83.1)	< 0.001	76.2 (68.0–84.5)	0.853	44/47	72.9 (65.3–80.4)	< 0.001	74.5 (67.1–81.9)	0.904

*AUC* Area under the curve, *H-L* Hosmer-Lemeshow, *NA* Not available, ^a^The equations for POSSUM and P-POSSUM are the same in the recalibration process

### Statistic analysis

Initially, we did a descriptive bivariate analysis of all the variables in the models in relation with in-hospital mortality and with 30-day mortality, using the chi-square test or Fisher’s exact test for categorical variables.

We validated the 5 predictive models, using the mathematical equations published by their creators (Additional file 1: Appendix A) and calculating the risk of operative mortality for each patient with the logistic regression link function.

Then multivariate logistic regression techniques were applied to recalibrate the 5 models, thus obtaining the new β coefficients for each score (POSSUM, P-POSSUM and CR-POSSUM models) or category of the variable (IRCS and AFC models). For these purposes, patients missing on any risk factor were excluded.

To calculate the discriminatory power of each model, we used receiver operating characteristic curves, calculating the areas under the curve (AUC) and their 95% confidence intervals. We considered an AUC between 70% and 80% moderate discrimination, between 80% and 90% good discrimination, and greater than 90% excellent [15]. We also estimated the calibration of the models by applying the Hosmer-Lemeshow test. We defined statistical significance as *p* < 0.05. We used IBM SPSS Statistics 20 and R 2.15.3 for all analyses.

## Results

A total of 3915 patients were recruited; 1166 (29.8%) were excluded for the reasons shown in Fig. 1. Thus, we analyzed 2749 patients (63.6% men; age range, 24–97 y; mean age, 68 ± 11 y) operated on for primary invasive colorectal cancer. The tumor was located in the colon in 1980 (72%) and in the rectum in 769 (28%) patients.

During hospital stay, 41 patients died (in-hospital operative mortality, 1.5% [95% CI: 1.0–1.9]) and 47 patients died within 30 days of the intervention (30-day operative mortality, 1.7% [95% CI: 1.2–2.2]).

Table 2 shows the variables in the CR-POSSUM in relation to in-hospital and 30-day mortality, as well as the summary of the physiological and operative severity scores. All the variables were significantly associated with in-hospital mortality and 30-day mortality, except heart rate, urea, and cancer stage, although stage was associated with 30-day mortality. Mortality was especially high in older patients, those with hypotension or heart failure, those undergoing urgent surgery, and those with free pus or faeces or blood. Additional file 1: Appendices B and C show the analysis of the factors used in the POSSUM, P-POSSUM, IRCS and AFC models. In the validation analysis, the discriminatory power of the CR-POSSUM for predicting in-hospital mortality was 73.6%, and the discriminatory power of the other models was similar (Table 3), although the number of patients with complete data as well as the number of deaths included in each model is different. When the models were recalibrated, the AUCs improved slightly (see Additional file 1: Appendix D and E for re-calibrated equations): the CR-POSSUM reached 75.5% (95% CI: 67.3–83.7) and the IRCS model had the highest discriminatory power with an AUC of 76.2 (95% CI: 68.0–84.5). The discriminatory power of the CR-POSSUM for predicting 30-day mortality was 74.2% (95% CI: 67.1–81.2) after recalibration; among the other recalibrated models the POSSUM had the greatest discriminatory power, with an AUC of 77.0% (95% CI: 68.9–85.2). Although the Hosmer-Lemeshow test showed good fit for all the recalibrated models, the original CR-POSSUM, as well as the original versions of the other models tended to overestimate the probability of operative death (Fig. 2).

## Discussion

In surgery for colorectal cancer, in-hospital mortality was 1.5% and 30-day mortality was 1.7%. The CR-POSSUM model, like the other validated models, overestimated operative mortality; once recalibrated, it had moderate discriminatory power as evidenced by the 75.5% AUC for in-hospital mortality and the 74.2% AUC for 30-day mortality.

### Operative mortality

The operative mortality observed in the present study is near the lower limits of the range of the estimations reported in similar studies [5, 16–26]. The 30-day mortality in these studies ranges from 0.7 and 11.3%. Various factors might have contributed to our low mortality rates. First, the proportion of patients undergoing urgent surgery in our study was low. Given that operative mortality is lower in scheduled than in urgent surgery, we would expect lower mortality in our series than in series with higher proportions of patients undergoing urgent surgery. Nevertheless, it is noteworthy that the operative mortality in the patients in our series that underwent urgent surgery was also lower than that reported in other previous studies. On the other hand, the patients in our study were operated on for a primary tumor in the period comprising 2010 through 2012, whereas most of the other studies discussed here examined earlier periods; thus, we cannot rule out a period effect involving a secular decrease in operative mortality for this kind of surgery over time due to various factors (e.g., improvements in perioperative management or different selection criteria for indication of surgery).

### Validity of CR-POSSUM and other POSSUM models

This validation and recalibration study of models for predicting operative mortality in a widespread, sample of Spanish hospitals found that the CR-POSSUM, has moderate discriminatory power, similar to that found in the external validation studies [8, 18, 25]. However, the original versions of this and the other models overestimated the operative mortality. To a certain extent, the low mortality observed in our cohort and the number of variables in the model limits our capacity to identify significant associations. It is worth mentioning that the model was designed to estimate in-hospital mortality, but we have seen that the AUC is similar for both types of mortality measure. On the other hand, this model was not designed solely for patients with colorectal cancer. One of the most widely questioned aspects of this model and of its predecessors, the POSSUM and P-POSSUM, is that some of the variables (operative variables) are not available until after the intervention; thus, they are not useful for predicting operative death. Another questionable aspect refers to the difficulties involved in obtaining all of the required variables (e.g., urea, staging), as we have observed in our study, despite its prospective design. For this reason, the other POSSUM models have similar limitations because they require collecting an even larger number of variables than the CR-POSSUM without resulting in appreciable improvements in their predictive capacity. In the bivariate analysis, some of the variables were not significantly associated with either in-hospital or 30-day mortality. Nevertheless, the discriminatory capacity of the three models was similar, considering their AUC and confidence intervals. In 2010, Richards et al. [27] reviewed the validation studies of these models, concluding that the P-POSSUM had the greatest discriminatory power of the three for colorectal cancer and that the CR-POSSUM, with an AUC < 75%, did not add any value, although a more recent external validation study reported better results [28]. It is therefore not surprising that several research teams have attempted to develop better models, given the contradictory results published before [29].

### Comparison with other models and with the literature

Of the models developed in Europe, the recently created IRCS model, which has fewer variables, yields a discriminatory capacity similar or even better than the POSSUM models; in our study, the IRCS predicted the outcome correctly in about three-quarters of the patients. In fact, of the models evaluated in this study, the IRCS is the one that best discriminated in-hospital operative mortality, although the POSSUM was slightly better at discriminating 30-day operative mortality. This difference might be related to the fact that advanced disease might not have as strong an impact on more immediate mortality as on longer-term mortality. Another advantage of the IRCS is the low number of variables, all of which, moreover, can be measured before the intervention, increasing the usefulness of the model for identifying patients at risk. None of the models used is specific for colorectal cancer; however, it might be that the more specific a model is for a particular disease or subgroup of patients (e.g., the elderly), the more complex its construction is, the lower its external validity, and the more difficult it will be to extend its use for clinical purposes or for assessment. This could explain why many of the models described in the literature have not had a great impact on clinical practice.

Very recently, a new model to predict in-hospital mortality in patients undergoing colorectal surgery, the Colorectal preOperative Surgical Score (CrOSS), was created and externally validated in Australia. Although it needs to be validated in other contexts, this model achieved an AUC of 0.87. It has the great advantage of considering only four variables, all of which can be assessed preoperatively (age, urgency of the intervention, albumin, and heart failure) [30]. The Association of Coloproctology of Great Britain and Ireland used multilevel analysis to devise a model specifically for predicting mortality risk in surgery for colorectal cancer, the ACPGBI-CRC. This model achieved an AUC of 77% [17]. Using one of the largest series of patients operated on for colorectal cancer, Walker et al. [24] devised a model that yielded an AUC of about 80% for estimating 90-day mortality. In this model, the predictor with the strongest association was the American Society of Anesthesiologists Physical Status classification (ASA grade), which itself has certain limitations in predicting surgical risk [31]. As for other predictive factors, advanced age is consistently associated with higher risk of death in the various models developed, as it is in many other health problems. Likewise, the urgency of the intervention, which reflects the patient’s condition, and certain underlying heart conditions are present in many of the models. Albumin or weight loss > 10% in the 6 months preceding surgery, both of which are indirect indicators of malnutrition before the intervention, also appear in different models. In fact, malnutrition is a clear risk factor for worse postoperative outcome in general, especially in older patients; it might also be the only factor considered in the models that can be modified before scheduled surgery.

The introduction of laparoscopic surgery in recent decades changes the scenario, and it is important to consider to what extent the lower risk of death reported in some studies [32] is independent of other factors. One of the most illustrative clinical trials found no differences in mortality between laparoscopic surgery and conventional open surgery [33]. In fact, most variables in the models are more related to the patient’s clinical condition than to the surgical technique used.

### Limitations

The cohort in this study includes a large series of patients recruited at 22 hospitals. As in all observational studies, the absence of information can be a limitation, although the prospective design and the quality control have enabled us to ensure thorough data collection. The missing data for some variables (e.g., some laboratory parameters) is due mostly to the unavailability of these factors in clinical practice, especially in the most urgent interventions. This made it impossible for us to use the entire sample of patients for some models; however, rather than a limitation due to the study design, this limitation is due to the models’ incompatibility with the available clinical information and/or routine clinical practice in our context. On the other hand, the mortality rate was low, with fewer than 50 deaths in both mortality indicators, and this might have compromised our capacity for recalibrating the models; however, in part thanks to the low mortality in our series, we were able to see that the original models considerably overestimated the risk of death.

### Clinical implications

This is the first multicenter study in Spain to validate and recalibrate some of the models for predicting operative mortality in a large cohort of patients operated on for colorectal cancer. Our data show that the operative mortality in these patients was low and that the models based on few variables that can be obtained in patients undergoing urgent surgery as well as those undergoing scheduled surgery can be useful in our healthcare system. Of the models we evaluated, the IRCS, which takes into account the patient’s age, the urgency of the intervention, the stage of disease, and the presence of respiratory failure or heart failure, is the one that might have the greatest discriminatory power for in-hospital mortality, although the POSSUM might be slightly better for predicting 30-day mortality. Nevertheless, there is considerable disparity in the factors that make up the models and none of them are generally used throughout Europe or in other areas, perhaps due to their moderate capacity to discriminate in the different contexts where they have been externally validated, as in our study. Our findings underline the need for a model that has better capacity to discriminate patients at greater risk; such a model should have face validity, be easy to apply, and be based on factors that can be measured before the intervention.

## Conclusions

The CR-POSSUM and the other models analyzed in this study showed moderate capability to discriminate the risk of operative mortality in our context, where the actual operative mortality is low. The IRCS model yielded similar results with fewer variables, all of which are available before the intervention. To optimize preoperative management and reduce operative mortality in patients undergoing surgery for colorectal cancer as far as possible, we need a model that can better discriminate the patients with greater risk.

## Additional file

Additional file 1: Appendix A. Equations for calculating risk of death for each predictive score. **Appendix B.** Univariate and bivariate analyses of POSSUM and P-POSSUM factors for operative mortality. **Appendix C.** Univariate and bivariate analyses of IRCS and AFC factors for operative mortality. **Appendix D.** Re-calibrated equations for calculating risk of in-hospital mortality for each predictive score. **Appendix E.** Re-calibrated equations for calculating risk of 30-day mortality for each predictive score. (DOCX 63 kb)

## Abbreviations

ACPGBI-CRC: Association of Coloproctology of Great Britain and Ireland - Colorectal Cancer
AFC: Association Française de Chirurgie
ASA grade: American Society of Anesthesiologists Physical Status classification
CrOSS: Colorectal preOperative Surgical Score
CR-POSSUM: Colorectal - Physiological and Operative Severity Score for the enUmeration of Mortality and Morbidity
IRCS: Identification of Risk in Colorectal Surgery
POSSUM: Physiological and Operative Severity Score for the enUmeration of Mortality and Morbidity
P-POSSUM: Portsmouth - Physiological and Operative Severity Score for the enUmeration of Mortality and Morbidity

## References

[1] International Agency for Research on Cancer. GLOBOCAN. Estimated Cancer Incidence, Mortality and Prevalence Worldwide in. 2012:2012. Available at: http://globocan.iarc.fr/Default.aspx. Accessed 8 June 2015

[2] Liang J, Fazio V, Lavery I, Remzi F, Hull T, Strong S (2015). Primacy of surgery for colorectal cancer. Br J Surg.

[3] Copeland GP, Jones D, Walters M (1991). POSSUM: a scoring system for surgical audit. Br J Surg.

[4] Prytherch DR, Whiteley MS, Higgins B, Weaver PC, Prout WG, Powell SJ (1998). POSSUM and Portsmouth POSSUM for predicting mortality. Physiological and operative severity score for the enUmeration of mortality and morbidity. Br J Surg.

[5] Tekkis PP, Prytherch DR, Kocher HM, Senapati A, Poloniecki JD, Stamatakis JD (2004). Development of a dedicated risk-adjustment scoring system for colorectal surgery (colorectal POSSUM). Br J Surg.

[6] Richards CH, Leitch FE, Horgan PG, McMillan DC (2010). A systematic review of POSSUM and its related models as predictors of post-operative mortality and morbidity in patients undergoing surgery for colorectal cancer. J Gastrointest Surg.

[7] Alves A, Panis Y, Mathieu P, Mantion G, Kwiatkowski F, Slim K, Association Française de Chirurgie (2005). Postoperative mortality and morbidity in French patients undergoing colorectal surgery: results of a prospective multicenter study. Arch Surg.

[8] van der Sluis FJ, Espin E, Vallribera F, de Bock GH, Hoekstra HJ, van Leeuwen BL (2014). Predicting postoperative mortality after colorectal surgery: a novel clinical model. Color Dis.

[9] Rodríguez-Cuellar E, Ruiz López P, Romero Simó M, Landa García JI, Roig Vila JV, Ortiz HH (2010). Analysis of the quality of surgical treatment of colorectal cancer, in 2008. A national study. Cir Esp.

[10] Espallargues M, Almazán C, Tebé C, Pla R, Pons JM, Sánchez E (2009). Management and outcomes in digestive cancer surgery: design and initial results of a multicenter cohort study. Rev Esp Enferm Dig.

[11] Valenti V, Hernandez-Lizoain JL, Baixauli J, Pastor C, Martinez-Regueira F, Beunza JJ (2009). Analysis of POSSUM score and postoperative morbidity in patients with rectal cancer undergoing surgery. Langenbeck's Arch Surg.

[12] Quintana JM, Gonzalez N, Anton-Ladislao A, Redondo M, Bare M, Fernandez de Larrea N, REDISSEC-CARESS/CCR group (2016). Colorectal cancer health services research study protocol: the CCR-CARESS observational prospective cohort project. BMC Cancer.

[13] Sobin LH, Gospodarowicz MK, Wittekind C (2009). The TNM classification of malignant Tumours 7th edition.

[14] The National Institute for Health and Care Exellence. Preoperative tests: The use of routine preoperative tests for elective surgery. NICE Clinical Guideline CG3; 2003. Available at: http://www.nice.org.uk/guidance/cg3. Accessed 10 June 2015.

[15] Harrell FE, Lee KL, Mark DB (1996). Multivariable prognostic models: issues in developing models, evaluating assumptions and adequacy, and measuring and reducing errors. Stat Med.

[16] Al-Refaie WB, Vickers SM, Zhong W, Parsons H, Rothenberger D, Habermann EB (2001). Cancer trials versus the real world in the United States. Ann Surg.

[17] Tekkis PP, Poloniecki JD, Thompson MR, Stamatakis JD (2003). Operative mortality in colorectal cancer: prospective national study. BMJ.

[18] Kong CH, Guest GD, Stupart DA, Faragher IG, Chan ST, Watters DA (2013). Recalibration and validation of a preoperative risk prediction model for mortality in major colorectal surgery. Dis Colon rectum.

[19] Morris EJ, Taylor EF, Thomas JD, Quirke P, Finan PJ, Coleman MP (2011). Thirty-day postoperative mortality after colorectal cancer surgery in England. Gut.

[20] Schootman M, Lian M, Pruitt SL, Hendren S, Mutch M, Deshpande AD (2014). Hospital and geographic variability in two colorectal cancer surgery outcomes: complications and mortality after complications. Ann Surg Oncol.

[21] van Erning FN, van Steenbergen LN, van den Broek WT, Rutten HJ, Lemmens VE (2013). No difference between lowest and highest volume hospitals in outcome after colorectal cancer surgery in the southern Netherlands. Eur J Surg Oncol.

[22] Henneman D, van Bommel AC, Snijders A, Snijders HS, Tollenaar RA, Wouters MW (2014). Ranking and rankability of hospital postoperative mortality rates in colorectal cancer surgery. Ann Surg.

[23] de Vries S, Jeffe DB, Davidson NO, Deshpande AD, Schootman M (2014). Postoperative 30-day mortality in patients undergoing surgery for colorectal cancer: development of a prognostic model using administrative claims data. Cancer Causes Control.

[24] Walker K, Finan PJ, van der Meulen JH (2015). Model for risk adjustment of postoperative mortality in patients with colorectal cancer. Br J Surg.

[25] Ramkumar T, Ng V, Fowler L, Farouk R (2006). A comparison of POSSUM, P-POSSUM and colorectal POSSUM for the prediction of postoperative mortality in patients undergoing colorectal resection. Dis Colon rectum.

[26] Ferjani AM, Griffin D, Stallard N, Wong LS (2007). A newly devised scoring system for prediction of mortality in patients with colorectal cancer: a prospective study. Lancet Oncol.

[27] Richards CH, Leitch EF, Anderson JH, McKee RF, McMillan DC, Horgan PG (2011). The revised ACPGBI model is a simple and accurate predictor of operative mortality after potentially curative resection of colorectal cancer. Ann Surg Oncol.

[28] Yan J, Wang Y-X, Li Z-P (2011). Predictive value of the POSSUM, p-POSSUM, CR-POSSUM, APACHEII and ACPGBI scoring systems in colorectal cancer resection. J Intern Med Res.

[29] Leung E, McArdle K, Wong LS (2011). Risk-adjusted scoring systems in colorectal surgery. Int J Surg.

[30] Kong CH, Guest GD, Stupart DA, Faragher IG, Chan ST, Watters DA (2015). Colorectal preOperative surgical score (CrOSS) for mortality in major colorectal surgery. ANZ J Surg.

[31] Protopapa KL, Simpson JC, Smith NC, Moonesinghe SR (2014). Development and validation of the surgical outcome risk tool (SORT). Br J Surg.

[32] Vallribera Valls F, Landi F, Espín Basany E, Sánchez García JL, Jiménez Gómez LM, Martí Gallostra M (2014). Laparoscopy-assisted versus open colectomy for treatment of colon cancer in the elderly: morbidity and mortality outcomes in 545 patients. Surg Endosc.

[33] Lacy AM, García-Valdecasas JC, Delgado S, Castells A, Taurá P, Piqué JM (2002). Laparoscopy-assisted colectomy versus open colectomy for treatment of non-metastatic colon cancer: a randomised trial. Lancet.

