# Supplementary material for: Validity of the CR-POSSUM model in surgery for colorectal cancer in Spain (CCR-CARESS study) and comparison with other models to predict operative mortality
Source: BMC Health Serv Res. 2018 Jan 29;18:49. doi: 10.1186/s12913-018-2839-x (PMC5789585; doi:10.1186/s12913-018-2839-x)
Supplement: Additional file 1: Appendix A. — Equations for calculating risk of death for each predictive score. Appendix B. Univariate and bivariate analyses of POSSUM and P-POSSUM factors for operative mortality. Appendix C. Univariate and bivariate analyses of IRCS and AFC factors for operative mortality. Appendix D. Re-calibrated equations for calculating risk of in-hospital mortality for each predictive score. Appendix E. Re-calibrated equations for calculating risk of 30-day mortality for each predictive score. (DOCX 63 kb) [file 12913_2018_2839_MOESM1_ESM.docx]

Appendix A. Equations for calculating risk of death for each predictive score

| Equations for calculating risk of death for each predictive score | | |  | |  |
| --- | --- | --- | --- | --- | --- |
| POSSUM | Ln [R/(1-R)] = -7.040 + (0.13 x physiological score) + (0.16 x operative score) | | | | |
| P-POSSUM | Ln [R/(1-R)] = -9.065 + (0.16 x physiological score) + (0.15 x operative score) | | | | |
| CR-POSSUM | Ln [R/(1-R)] = -9.167 + (0.33 x physiological score) + (0.30 x operative score) | | | | |
| IRCS | Ln [R/(1-R)] = -5.526 + (0.484 x age 61-70) | |  | |  |
|  |  | + (1.181 x age 71-80) |  | |  |
|  |  | + (1.934 x age >80)) |  | |  |
|  |  | + (1.317 x tumour stage) |  | |  |
|  |  | + (2.027 x urgent surgery) | |  |  |
|  |  | + (0.903 x heart failure) |  | |  |
|  |  | + (1.207 x respiratory failure) | | |  |
| ^R. predicted risk of death for CR-POSSUM, POSSUM, P-POSSUM predictive scores and for IRCS score.^ | | | | | |

Appendix B. Univariate and bivariate analyses of POSSUM and P-POSSUM factors for operative mortality.

|  | |  | |  | Total (N=2749) | | | | In-hospital mortality (N=41) | | | | | | 30-day mortality (N=47) | | | | | |  |  |
| --- | --- | --- | --- | --- | --- | --- | --- | --- | --- | --- | --- | --- | --- | --- | --- | --- | --- | --- | --- | --- | --- | --- |
|  | | Weight | |  | N | | %Col | | N | | %Row | | p-value | | N | | %Row | | p-value | |  |  |
| Age | | 1 | | <61 | 640 | | 23.3 | | 2 | | 0.3 | | **<0.001** | | 2 | | 0.3 | | **<0.001** | |  |  |
|  |  | 2 | | 61 - 70 | 830 | | 30.2 | | 7 | | 0.8 | |  |  | 10 | | 1.2 | |  |  |  |  |
|  |  | 4 | | >70 | 1275 | | 46.4 | | 32 | | 2.5 | |  |  | 35 | | 2.7 | |  |  |  |  |
|  |  |  | | *missing* | *4* | | *0.1* | |  | |  | |  | |  | |  | |  | |  |  |
| Heart failure | | 1 | | None | 2083 | | 77.8 | | 25 | | 1.2 | | **0.001** | | 28 | | 1.3 | | **0.001** | |  |  |
|  |  | 2 | | Mild | 404 | | 15.1 | | 6 | | 1.5 | |  |  | 8 | | 2.0 | |  |  |  |  |
|  |  | 4 | | Moderate | 144 | | 5.4 | | 6 | | 4.2 | |  |  | 7 | | 4.9 | |  |  |  |  |
|  |  | 8 | | Severe | 45 | | 1.7 | | 3 | | 6.7 | |  |  | 3 | | 6.7 | |  |  |  |  |
|  |  |  | | *missing* | *73* | | *2.7* | |  | |  | |  | |  | |  | |  | |  |  |
| Respiratory failure | | 1 | | No dyspnoea | 2360 | | 88.1 | | 22 | | 0.9 | | **<0.001** | | 26 | | 1.1 | | **<0.001** | |  |  |
|  |  | 2 | | Dyspnoea on exertion | 231 | | 8.6 | | 10 | | 4.3 | |  |  | 12 | | 5.2 | |  |  |  |  |
|  |  | 4 | | Limiting dyspnoea | 84 | | 3.1 | | 5 | | 6.0 | |  |  | 6 | | 7.1 | |  |  |  |  |
|  |  | 8 | | Dyspnoea at rest | 5 | | 0.2 | | 1 | | 20.0 | |  |  | 1 | | 20.0 | |  |  |  |  |
|  |  |  | | *missing* | *69* | | *2.5* | |  | |  | |  | |  | |  | |  | |  |  |
| Systolic blood pressure (mmHg) | | 1 | | 110 - 130 | 1023 | | 38.9 | | 15 | | 1.5 | | **0.012** | | 15 | | 1.5 | | **0.017** | |  |  |
|  |  | 2 | | 131 - 170 or 100 - 109 | 1429 | | 54.3 | | 19 | | 1.3 | |  |  | 24 | | 1.7 | |  |  |  |  |
|  |  | 4 | | ≥171 or 90 - 99 | 161 | | 6.1 | | 4 | | 2.5 | |  |  | 5 | | 3.1 | |  |  |  |  |
|  |  | 8 | | ≤89 | 20 | | 0.8 | | 2 | | 10.0 | |  |  | 2 | | 10.0 | |  |  |  |  |
|  |  |  | | *missing* | *116* | | *4.2* | |  | |  | |  | |  | |  | |  | |  |  |
| Heart rate (beats/min) | | 1 | | 50 - 80 | 1835 | | 70.4 | | 24 | | 1.3 | | 0.255 | | 25 | | 1.4 | | 0.100 | |  |  |
|  |  | 2 | | 81 - 100 or 40 - 49 | 681 | | 26.1 | | 12 | | 1.8 | |  |  | 17 | | 2.5 | |  |  |  |  |
|  |  | 4 | | 101 - 120 | 76 | | 2.9 | | 3 | | 3.9 | |  |  | 3 | | 3.9 | |  |  |  |  |
|  |  | 8 | | ≥121 or ≤39 | 13 | | 0.5 | | 0 | | 0.0 | |  |  | 0 | | 0.0 | |  |  |  |  |
|  |  |  | | *missing* | *144* | | *5.2* | |  | |  | |  | |  | |  | |  | |  |  |
| Glasgow coma score | | 1 | | 15 | 2745 | | 99.9 | | 41 | | 1.5 | | 0.862 | | 47 | | 1.7 | | 0.852 | |  |  |
|  |  | 2 | | 12 - 14 | 2 | | 0.1 | | 0 | | 0.0 | |  |  | 0 | | 0.0 | |  |  |  |  |
|  |  | 4 | | 9 - 11 | 0 | | 0.0 | | 0 | | 0.0 | |  |  | 0 | | 0.0 | |  |  |  |  |
|  |  | 8 | | ≤8 | 0 | | 0.0 | | 0 | | 0.0 | |  |  | 0 | | 0.0 | |  |  |  |  |
|  |  |  | | *missing* | *2* | | *0.1* | |  | |  | |  | |  | |  | |  | |  |  |
| Haemoglobin (g/dl) | | 1 | | 13.0 - 16.0 | 1053 | | 39.1 | | 13 | | 1.2 | | **0.045** | | 12 | | 1.1 | | **0.004** | |  |  |
|  |  | 2 | | 11.5 - 12.9 or 16.1 - 17.0 | 769 | | 28.6 | | 8 | | 1.0 | |  |  | 11 | | 1.4 | |  |  |  |  |
|  |  | 4 | | 10.0 - 11.4 or 17.1 - 18.0 | 521 | | 19.3 | | 8 | | 1.5 | |  |  | 10 | | 1.9 | |  |  |  |  |
|  |  | 8 | | ≤9.9 or ≥18.1 | 350 | | 13.0 | | 11 | | 3.1 | |  |  | 14 | | 4.0 | |  |  |  |  |
|  |  |  | | *missing* | *56* | | *2.0* | |  | |  | |  | |  | |  | |  | |  |  |
| Leucocytes  (x 10^12 /l) | | 1 | | 4.0 - 10.0 | 2228 | | 83.0 | | 28 | | 1.3 | | **0.011** | | 32 | | 1.4 | | **0.001** | |  |  |
|  |  | 2 | | 10.1 - 20.0 or 3.1 - 3.9 | 406 | | 15.1 | | 9 | | 2.2 | |  |  | 11 | | 2.7 | |  |  |  |  |
|  |  | 4 | | ≥20.1 or ≤3.0 | 51 | | 1.9 | | 3 | | 5.9 | |  |  | 4 | | 7.8 | |  |  |  |  |
|  |  |  | | *missing* | *64* | | *2.3* | |  | |  | |  | |  | |  | |  | |  |  |
| Urea (mmol/l) | | 1 | | ≤7.5 | 329 | | 13.3 | | 5 | | 1.5 | | 0.575 | | 5 | | 1.5 | | 0.455 | |  |  |
|  |  | 2 | | 7.6 - 10.0 | 438 | | 17.8 | | 9 | | 2.1 | |  |  | 10 | | 2.3 | |  |  |  |  |
|  |  | 4 | | 10.1 - 15.0 | 1005 | | 40.8 | | 12 | | 1.2 | |  |  | 15 | | 1.5 | |  |  |  |  |
|  |  | 8 | | ≥15.1 | 694 | | 28.1 | | 13 | | 1.9 | |  |  | 17 | | 2.4 | |  |  |  |  |
|  |  |  | | *missing* | *283* | | *10.3* | |  | |  | |  | |  | |  | |  | |  |  |
|  | | | | | | | Total (N=2749) | | | | In-hospital mortality (N=41) | | | | | | 30-day mortality (N=47) | | | | | |
|  | | | | | | | N | | %Col | | N | | %Row | | p-value | | N | | %Row | | p-value | |
| Sodium (mmol/l) | | 1 | | ≥136 | | | 2488 | | 95.1 | | 38 | | 1.5 | | 0.077 | | 43 | | 1.7 | | 0.111 | |
|  |  | 2 | | 131 - 135 | | | 110 | | 4.2 | | 1 | | 0.9 | |  |  | 3 | | 2.7 | |  |  |
|  |  | 4 | | 126 - 130 | | | 8 | | 0.3 | | 1 | | 12.5 | |  |  | 1 | | 12.5 | |  |  |
|  |  | 8 | | ≤125 | | | 11 | | 0.4 | | 0 | | 0.0 | |  |  | 0 | | 0.0 | |  |  |
|  |  |  | | *missing* | | | *132* | | *4.8* | |  | |  | |  | |  | |  | |  | |
| Potassium (mmol/l) | | 1 | | 3.5 - 5.0 | | | 2309 | | 88.7 | | 31 | | 1.3 | | **0.046** | | 37 | | 1.6 | | 0.104 | |
|  |  | 2 | | 3.2 - 3.4 or 5.1 - 5.3 | | | 172 | | 6.6 | | 3 | | 1.7 | |  |  | 4 | | 2.3 | |  |  |
|  |  | 4 | | 2.9 - 3.1 or 5.4 - 5.9 | | | 94 | | 3.6 | | 3 | | 3.2 | |  |  | 3 | | 3.2 | |  |  |
|  |  | 8 | | ≤2.8 or ≥6.0 | | | 29 | | 1.1 | | 2 | | 6.9 | |  |  | 2 | | 6.9 | |  |  |
|  |  |  | | *missing* | | | *145* | | *5.3* | |  | |  | |  | |  | |  | |  | |
| Electrocardiogram | | 1 | | Normal | | | 2205 | | 82.7 | | 24 | | 1.1 | | **<0.001** | | 28 | | 1.3 | | **<0.001** | |
|  |  | 4 | | Atrial fibrillation | | | 264 | | 9.9 | | 12 | | 4.5 | |  |  | 13 | | 4.9 | |  |  |
|  |  | 8 | | Other abnormal rhythm | | | 198 | | 7.4 | | 3 | | 1.5 | |  |  | 3 | | 1.5 | |  |  |
|  |  |  | | *missing* | | | *82* | | *3.0* | |  | |  | |  | |  | |  | |  | |
| **Physiological score** | | | | | | | **mean: 21.8** | | **std. dev: 5.5** | | **median: 21.0** | | **min: 12.0** | | **max: 47.0** | | *missing: 508* | |  | |  | |
| Operative severity | | 1 | | Minor | | | 0 | | 0.0 | | 0 | | 0.0 | | **0.003** | | 0 | | 0.0 | | **<0.001** | |
|  |  | 2 | | Moderate | | | 60 | | 2.2 | | 4 | | 6.7 | |  |  | 5 | | 8.3 | |  |  |
|  |  | 4 | | Major | | | 1520 | | 55.4 | | 19 | | 1.2 | |  |  | 21 | | 1.4 | |  |  |
|  |  | 8 | | Complex Major | | | 1164 | | 42.4 | | 18 | | 1.5 | |  |  | 21 | | 1.8 | |  |  |
|  |  |  | | *missing* | | | *5* | | *0.2* | |  | |  | |  | |  | |  | |  | |
| Multiple procedures | | 1 | | 1 | | | 144 | | 5.3 | | 3 | | 2.1 | | 0.809 | | 3 | | 2.1 | | 0.925 | |
|  |  | 4 | | 2 | | | 1583 | | 57.8 | | 24 | | 1.5 | |  |  | 26 | | 1.6 | |  |  |
|  |  | 8 | | >2 | | | 1010 | | 36.9 | | 14 | | 1.4 | |  |  | 17 | | 1.7 | |  |  |
|  |  |  | | *missing* | | | *12* | | *0.4* | |  | |  | |  | |  | |  | |  | |
| Total blood loss (ml) | | 1 | | ≤100 | | | 2146 | | 82.3 | | 19 | | 0.9 | | **<0.001** | | 28 | | 1.3 | | **0.002** | |
|  |  | 2 | | 101 - 500 | | | 333 | | 12.8 | | 6 | | 1.8 | |  |  | 5 | | 1.5 | |  |  |
|  |  | 4 | | 501 - 999 | | | 74 | | 2.8 | | 2 | | 2.7 | |  |  | 2 | | 2.7 | |  |  |
|  |  | 8 | | ≥1.000 | | | 53 | | 2.0 | | 5 | | 9.4 | |  |  | 4 | | 7.5 | |  |  |
|  |  |  | | *missing* | | | *143* | | *5.2* | |  | |  | |  | |  | |  | |  | |
| Peritoneal contamination | | 1 | | None | | | 2615 | | 95.5 | | 36 | | 1.4 | | **0.003** | | 39 | | 1.5 | | **<0.001** | |
|  |  | 2 | | Serous fluid | | | 68 | | 2.5 | | 1 | | 1.5 | |  |  | 3 | | 4.4 | |  |  |
|  |  | 4 | | Local pus | | | 9 | | 0.3 | | 1 | | 11.1 | |  |  | 1 | | 11.1 | |  |  |
|  |  | 8 | | Free pus or faeces or blood | | | 46 | | 1.7 | | 3 | | 6.5 | |  |  | 4 | | 8.7 | |  |  |
|  |  |  | | *missing* | | | *11* | | *0.4* | |  | |  | |  | |  | |  | |  | |
| Presence of malignancy | | 1 | | None | | | 0 | | 0.0 | | 0 | | 0.0 | | 0.484 | | 0 | | 0.0 | | **0.003** | |
|  |  | 2 | | Primary only | | | 1582 | | 57.9 | | 20 | | 1.3 | |  |  | 21 | | 1.3 | |  |  |
|  |  | 4 | | Nodal metastases | | | 895 | | 32.7 | | 16 | | 1.8 | |  |  | 15 | | 1.7 | |  |  |
|  |  | 8 | | Distant metastases | | | 256 | | 9.4 | | 5 | | 2.0 | |  |  | 11 | | 4.3 | |  |  |
|  |  |  | | *missing* | | | *16* | | *0.6* | |  | |  | |  | |  | |  | |  | |
| Operative urgency | | 1 | | Scheduled | | | 2649 | | 96.4 | | 34 | | 1.3 | | **<0.001** | | 38 | | 1.4 | | **<0.001** | |
|  |  | 4 | | Urgent | | | 93 | | 3.4 | | 6 | | 6.5 | |  |  | 8 | | 8.6 | |  |  |
|  |  | 8 | | Emergency | | | 7 | | 0.3 | | 1 | | 14.3 | |  |  | 1 | | 14.3 | |  |  |
|  |  |  | | *missing* | | | *0* | | *0.0* | |  | |  | |  | |  | |  | |  | |
| **Operative severity score** | | | | | | | **mean: 17.8** | | **std. dev: 4.7** | | **median: 17.0** | | **min: 8.0** | | **max: 40.0** | | *missing: 179* | |  | |  | |

Appendix C. Univariate and bivariate analyses of IRCS and AFC factors for operative mortality.

|  |  |  | Total (N=2749) | | In-hospital mortality (N=41) | | | 30-day mortality (N=47) | | |
| --- | --- | --- | --- | --- | --- | --- | --- | --- | --- | --- |
| **IRCS** | Weight |  | N | %Col | N | %Row | p-value | N | %Row | p-value |
| Age | 0 | ≤60 | 640 | 23.3 | 2 | 0.3 | **<0.001** | 2 | 0.3 | **<0.001** |
|  | 1 | 61 - 70 | 830 | 30.2 | 7 | 0.8 |  | 10 | 1.2 |  |
|  | 2 | 71 - 80 | 902 | 32.9 | 14 | 1.6 |  | 16 | 1.8 |  |
|  | 3 | ≥81 | 373 | 13.6 | 18 | 4.8 |  | 19 | 5.1 |  |
|  |  | *Missing* | *4* | *0.1* |  |  |  |  |  |  |
| Tumour stage | 0 | Stage I/II | 1582 | 57.9 | 20 | 1.3 | 0.234 | 21 | 1.3 | 0.064 |
|  | 1 | Stage III/IV | 1151 | 42.1 | 21 | 1.8 |  | 26 | 2.3 |  |
|  |  | *Missing* | *16* | *0.6* |  |  |  |  |  |  |
| Urgent surgery | 0 | No | 2649 | 96.4 | 34 | 1.3 | **0.001** | 38 | 1.4 | **<0.001** |
|  | 2 | Yes | 100 | 3.6 | 7 | 7.0 |  | 9 | 9.0 |  |
|  |  | *Missing* | *0* | *0.0* |  |  |  |  |  |  |
| Heart failure | 0 | None or mild | 2487 | 92.9 | 31 | 1.2 | **0.001** | 36 | 1.4 | **0.001** |
|  | 1 | Moderate or severe | 189 | 7.1 | 9 | 4.8 |  | 10 | 5.3 |  |
|  |  | *Missing* | *73* | *2.7* |  |  |  |  |  |  |
| Respiratory failure | 0 | None or mild | 2591 | 96.7 | 32 | 1.2 | **0.001** | 38 | 1.5 | **0.001** |
|  | 1 | Moderate or severe | 89 | 3.3 | 6 | 6.7 |  | 7 | 7.9 |  |
|  |  | *Missing* | *69* | *2.5* |  |  |  |  |  |  |
| **AFC** | | | | | | | | | | |
| Age > 70 yr |  | No | 1470 | 53.6 | 9 | 0.6 | **<0.001** | 12 | 0.8 | **<0.001** |
|  |  | Yes | 1275 | 46.4 | 32 | 2.5 |  | 35 | 2.7 |  |
|  |  | *Missing* | *4* | *0.1* |  |  |  |  |  |  |
| Weight loss >10% |  | No | 1326 | 90.5 | 18 | 1.4 | 0.061 | 20 | 1.5 | **0.031** |
|  |  | Yes | 140 | 9.5 | 5 | 3.6 |  | 6 | 4.3 |  |
|  |  | *Missing* | *1283* | *46.7* |  |  |  |  |  |  |
| Neurological comorbidities |  | No | 2593 | 94.5 | 33 | 1.3 | **0.001** | 39 | 1.5 | **0.004** |
|  |  | Yes | 152 | 5.5 | 8 | 5.3 |  | 8 | 5.3 |  |
|  |  | *Missing* | *4* | *0.1* |  |  |  |  |  |  |
| Emergency surgery |  | No | 2585 | 94.0 | 36 | 1.4 | 0.094 | 38 | 1.5 | **0.001** |
|  |  | Yes | 164 | 6.0 | 5 | 3.0 |  | 9 | 5.5 |  |
|  |  | *Missing* | *0* | *0.0* |  |  |  |  |  |  |

Appendix D. Re-calibrated equations for calculating risk of in-hospital mortality for each predictive score

| Equations for calculating risk of in-hospital mortality for each predictive score | | | | | |
| --- | --- | --- | --- | --- | --- |
| POSSUM &  P-POSSUM | Ln [R/(1-R)] = -9.441 + (0.122 x physiological score) + (0.114 x operative score) | | | | |
| CR-POSSUM | Ln [R/(1-R)] = -8.972 + (0.298 x physiological score) + (0.148 x operative score) | | | | |
| IRCS | Ln [R/(1-R)] = -5.983 + (0.962 x age 61-70) | |  | |  |
|  |  | + (0.679 x age 71-80) |  | |  |
|  |  | + (0.826 x age >80)) |  | |  |
|  |  | + (0.153 x tumour stage) |  | |  |
|  |  | + (0.805 x urgent surgery) | |  |  |
|  |  | + (0.628 x heart failure) |  | |  |
|  |  | + (1.147 x respiratory failure) | | |  |
| AFC | Ln [R/(1-R)] = -5.086 + (1.141 x Age > 70 yr) | | | | |
|  | + (1.027 x Weight loss >10%) | | | | |
|  | + (0.774 x Neurological comorbidities) | | | | |
|  | + (0.447 x Emergency surgery) | | | | |
| ^R. predicted risk of death for CR-POSSUM, POSSUM, P-POSSUM predictive scores and for IRCS and AFC score.^ | | | | | |

Appendix E. Re-calibrated equations for calculating risk of 30-day mortality for each predictive score

| Equations for calculating risk of 30-day mortality for each predictive score | | | | | |
| --- | --- | --- | --- | --- | --- |
| POSSUM &  P-POSSUM | Ln [R/(1-R)] = -9.447 + (0.126 x physiological score) + (0.118 x operative score) | | | | |
| CR-POSSUM | Ln [R/(1-R)] = -8.951 + (0.287 x physiological score) + (0.178 x operative score) | | | | |
| IRCS | Ln [R/(1-R)] = -6.105 + (1.330 x age 61-70) | |  | |  |
|  |  | + (0.798 x age 71-80) |  | |  |
|  |  | + (0.826 x age >80)) |  | |  |
|  |  | + (0.366 x tumour stage) |  | |  |
|  |  | + (0.802 x urgent surgery) | |  |  |
|  |  | + (0.646 x heart failure) |  | |  |
|  |  | + (1.224 x respiratory failure) | | |  |
| AFC | Ln [R/(1-R)] = -4.978 + (1.122 x Age > 70 yr) | | | | |
|  | + (1.096 x Weight loss >10%) | | | | |
|  | + (0.649 x Neurological comorbidities) | | | | |
|  | + (0.772 x Emergency surgery) | | | | |
| ^R. predicted risk of death for CR-POSSUM, POSSUM, P-POSSUM predictive scores and for IRCS and AFC score.^ | | | | | |
